# Association of Certificated Blind Masseurs

**Published:** 1920-02-28

**Authors:** 


### Association of Certificated Blind Masseurs.

The above Association was registered on July 3, 1919, and licensed under the Board of Trade. The objects for which the Association is established are :

"To promote the welfare and protect and advance the interests of all certificated and qualified masseurs and masseuses who are too blind to perform work for which eyesight is essential."

"To assist and secure the recognition and status of such blind masseurs as aforesaid in and for the purposes of their work."

"To provide for such blind masseurs any assistance or advantage calculated to help them to work on terms of equality with sighted masseurs."

"To promote co-operation in all matters relating to massage and physical culture between the blind and sighted persons."

"To advocate and extend the general use or employment of massage and other physical methods of exercises."

### Qualification of Members.

To be eligible for membership of the Association it is essential for a blind masseur to hold the certificate of the Incorporated Society of Trained Masseuses or the certificate of the late Dr. Fletcher Little, during that gentleman's lifetime, or such other certificate as may, from time to time, be prescribed by the Executive Council. The President of the Association is Sir Arthur Pearson, Bart., G.B.E., and amongst the list of well-known medical men who are Vice-Presidents appear the names of Sir Bruce Bruce-Porter, K.B.E., C.M.G., Sir Alfred Fripp, K.C.V.O., C.B., Major-General Sir Robert Jones, K.B.E., C.B., Sir Alfred Pearce Gould, K.C.V.O., Dr. Risien Russell, Major W. H. Broad, etc., etc. The control and management of the business of the Association are vested in an Executive Council, consisting exclusively of members of the Association. The Chairman for the present year is Mr. Percy Linney Way, who is well known in the massage world. The registered office of the Association of Certificated Blind Masseurs is 224-6-8 Great Portland Street, London, W., where application for masseurs may be made to the Secretary. (Telephone: Mayfair 4102-3-4.)

